# Exploring Obstetrical Interventions and Stratified Cesarean Section Rates Using the Robson Classification in Tertiary Care Hospitals in the United Arab Emirates

**DOI:** 10.1055/s-0038-1676524

**Published:** 2019-03-14

**Authors:** Mahera Abdulrahman, Sara Saad Abdullah, Aminah Fuad Khalil Alaani, Noora Hassan AlAbdool, Fatma Elzahraa Yehia Sherif, Zainab SalahEldin Ahmed, Hiba Issa Al-Rawi, Nawal Mahmood Hubaishi, Muna AbdulRazzaq Tahlak, Frederick R Carrick

**Affiliations:** 1 Department of Medical Education and Research, Dubai Health Authority, Dubai, United Arab Emirates; 2 Department of Primary Health Care, Dubai Medical College, Dubai, United Arab Emirates; 3 Dubai Health Authority, Latifa Hospital, Dubai, United Arab Emirates; 4 Dubai Health Authority, Dubai Hospital, Dubai, United Arab Emirates; 5 Bedfordshire Centre for Mental Health Research in association with University of Cambridge, Cambridge, United Kingdom; 6 Harvard Medical School-Harvard Macy Institute, Boston, MA, United States; 7 Neurology Department, Carrick Institute, Cape Canaveral, FL, United States

**Keywords:** obstetrical interventions, cesarean section rate, robson classification, united arab emirates, women

## Abstract

**Objective** The objective of the present study was to explore obstetric management in relation to clinical, maternal and child health outcomes by using the Robson classification system.

**Methods** Data was collected from obstetrics registries in tertiary care hospitals in Dubai, United Arab Emirates (UAE).

**Results** The analysis of > 5,400 deliveries (60% of all the deliveries in 2016) in major maternity hospitals in Dubai showed that groups 5, 8 and 9 of Robson’s classification were the largest contributors to the overall cesarean section (CS) rate and accounted for 30% of the total CS rate. The results indicate that labor was spontaneous in 2,221 (45%) of the women and was augmented or induced in almost 1,634 cases (33%). The birth indication rate was of 64% for normal vaginal delivery, of 24% for emergency CS, and of 9% for elective CS. The rate of vaginal birth after cesarean was 261 (6%), the rate of external cephalic version was 28 (0.7%), and the rate of induction was 1,168 (21.4%). The prevalence of the overall Cesarean section was 33%; with majority (53.5%) of it being repeated Cesarean section.

**Conclusion** The CS rate in the United Arab Emirates (UAE) is higher than the global average rate and than the average rate in Asia, which highlights the need for more education of pregnant women and of their physicians in order to promote vaginal birth. A proper planning is needed to reduce the number of CSs in nulliparous women in order to prevent repeated CSs in the future. Monitoring both CS rates and outcomes is essential to ensure that policies, practices, and actions for the optimization of the utilization of CS lead to improved maternal and infant outcomes.

## Introduction

The crude rate of cesarean section (CS) deliveries is considered an important global indicator when measuring the access to obstetric care.^1^ In 1985, the World Health Organization (WHO) stated that there was no justification for any region to have a CS rate higher than 10 to 15%.^2^ However, this rate has been increased over the last two decades; especially in middle- and high-income countries.^3^ The reason behind increased CS rates is considered multifactorial, with contributions from both medical factors, such as increase of high-risk pregnancies^4^ and preterm deliveries^5^, and psychosocial factors, such as a CS on demand.^6^ ^7^

It is well known that CS carry its own risks for maternal and infant morbidity and for subsequent pregnancies.^8^ Therefore, the rise in CS rates is becoming a major public health concern, and the factors that are causing this phenomenon, as well as the strategies to reduce CS rates, are intensively analyzed.^9^ ^10^ However, to propose and implement effective measures to reduce the CS rates, it is first essential to identify which groups of women are undergoing CS and to investigate the underlying reasons in different settings. The Robson 10-group classification system is one of the best methods that fulfills the current international and institutional needs to monitor and analyze CS rates.^1^ Applying the Robson classification to the data should allow the identification of the subgroup(s) that are predominantly contributing to the steady increase in the overall CS rate.

The United Arab Emirates (UAE) is a young country with families tending to have large numbers of children. A recent publication by Tahlak et al showed that the CS rate has increased in the past 15 years from 1 in every 5 births to 1 in every 3 births.^11^ New insurance policies have encouraged the private sectors to develop a more sophisticated management that may be associated with an increase in health care costs.

The increase in multiple pregnancies and in the CS rate has led to an increased rate of hysterectomy and of other obstetrical complications. Nowadays, it is common for any obstetrician working in the UAE to come across and manage pregnant women who have had more than three CSs. The increase of obstetrical complications has resulted in a burdensome government health system that desires to encourage high birth rates, but at the same time to decrease birth complications. The purpose of the present study is to evaluate obstetrics management in governmental tertiary hospitals in Dubai, UAE. We aim to assess the current obstetrics management in relation to clinical and maternal and child health outcomes, in order to determine whether the increase in CS rates is genuinely due to changes in patient epidemiology and in risk factors or merely due to changes in obstetric management.

## Methods

### Data Source and Study Variables

Information on all deliveries that occurred in the Dubai Health Authority (DHA) between January 1^st^, 2016, until September 30^th^, 2016, was accessed from delivery registries of hospitals in Latifa and in Dubai. A well designed questionnaire form was used to collect clinical data and information on maternal medical conditions, labor and delivery events, neonatal outcomes, and other maternal characteristics. The maternal characteristics included maternal age, nationality, parity, and gestational age. Data were also collected for maternal conditions or diseases, such as diabetes, hypertensive disorders during pregnancy, and history of previous CS. Upon acquisition, the data was sorted according to the Robson 10-group classification system.^12^

The outcome of the pregnancy was categorized by induction use, and its indication, augmentation, interventions and rupture of the membrane. Information about the route of delivery (normal vaginal delivery [NVD], forceps, vacuum, emergency CS, elective CS), the type of anesthesia used, the condition of the perineum, that is, whether the delivery was associated with tears or episiotomy, along with its degree, and whether the patient lost blood and received a blood transfusion, were also included. The following complications were also documented: antepartum and postpartum hemorrhage, abruption, placenta previa, shoulder dystocia, cord prolapse, hysterectomy, ruptured uterus, and tubal ligation. In addition, information was documented about the position of the fetus and about the status that identified whether the fetus had any anomaly, scalp injury, or intrauterine growth retardation, or was classified as an intrauterine death. In addition, it was recorded whether the delivery was performed by an obstetrician or a midwife. We suggest that changes in the maternal characteristics (e.g., increases in the maternal age), as well as maternal conditions or diseases (e.g., multifetal pregnancy) can lead to changes in the obstetric practice (e.g., increases in the induction rates). Therefore, a sequential model was used to identify the effect of each factor and of each group of elements. Finally, we have adjusted for fetal or infant characteristics, including gestational age, small for gestational age, and birth weight.

### Outcome Measures and Other Variables

The primary outcome was the intrapartum CS rate. Vaginal instrumental (vacuum or forceps) birth, pharmacological sedation or analgesia, epidural anesthesia, and augmentation of labor with oxytocin were secondary outcomes.

### Data Analysis and Statistics

All of the collected data were entered into SPSS Statistics for Windows, Version 21.0 (IBM Corp., Armonk, NY, USA) for statistical analysis. Descriptive statistics were computed for the sociodemographic variables. The overall data was recorded as a percentage of the total. The differences were determined using the Chi-squared test, and the statistical significance was recorded for non-parametric data. We fit several multiple regression models with an α of 0.05 and a power of 80% to better understand the predictability of obstetrical interventions and outcomes. The potential determinants of primary CS were categorized into several groups: maternal characteristics, maternal conditions or diseases, factors related to obstetric practice, and fetal or infant characteristics. All of the analyses were conducted with the SPSS Statistics for Windows, Version 20.0 (IBM Corp., Armonk, NY, USA). The semipartial correlation in multiple linear regression was calculated using the Stata Statistical Software: Release 14 (Statacorp, College Station, TX, USA). The semipartial correlation (semipartial R^2^) in multiple linear regression was calculated using STATA version 14, College Station, Texas 77845 USA.

### Ethics Statement

The present study was approved by the institutional review board of the DHA, Dubai (DSREC-08/2016_07). The aggregate reporting of data and coding assured to enhance the confidentiality of information.

## Results

A total of 5,461 pregnancies were recorded with a gestational age from 24 to 42 weeks. Table 1 shows the characteristics of all births/pregnancies. The majority (2,374; 43.5%) of the women was between 20 and 29 years old, UAE nationals (2,941; 54%), having previous 1 to 4 parities (3,536; 65%), with gestational age of 37 to 41 weeks (4,498; 82%), and booked with the hospital for regular visits (5,060; 93%). A total of 1,291 (24%) women had a history of previous CS, of which 78 (2%) had more than 3 previous CSs. The labor was spontaneous in 2,221 (45%) women, and augmented or induced in 1,634 (33%) (Table 1).

**Table 1:** Demographic and clinical characteristics of the mothers, birth indication, and complications

Characteristic		All Women *n* = 5461 (n; %)		
Maternal Age (years old)	< 20 (76; 1.5%)	20–29 (2,361; 43.5%)	30–34 (1,673; 31%)	≥35 (1,300; 24%)
Nationality	UAE national (2,923; 54%)	Non-UAE national (2,504; 46%)		
Parity	0 (1,370; 25%)	1–4 (3,516; 65%)	> 4 (535; 10%)	
Gestational age (weeks)	24–28 (65; 1%)	28–36 (807; 15%)	37–41 (4,498; 82%)	≥41 (91; 2%)
Diabetes	None (4,345; 79%)	IDDM (80; 2%)	NIDDM (43; 1%)	Gestational (986; 18%)
Hypertension	None (5,092; 94%)	High BP (263; 5%)	Preeclamptic (73; 1%)	
Previous CS	None (849; 46.5%)	1 (474; 26%)	2 (273; 15%)	3 (151; 8.3%); > 3 (77; 4.2%)
Booked	Yes (5,060; 93%)	No (401; 7%)		
Vaginal birth after cesarean	Yes (261; 6%)			
External cephalic version	Yes (28; 0.7%)			
Birth indication	NVD (3,422; 63%)	Forceps (12; 0.2%)	Vacuum (170; 3%)	Emergency CS (1,316; 24%) Elective CS (508; 9%)
Anesthesia	None (3,655; 67%)	Spinal (1,307; 24%)	General (361; 7%)	Epidural (138; 2%)
Perineum	Intact (2,727; 53%)	1^°^ (790; 15%)	2^°^ (484; 9%)	3 or 4^°^ (40; 1%)
	Episiotomy (983; 19%)	Multiple tears (24; 0.5%)	Cervical (22; 0.5%)	Para urethral (81; 2%)
Induction	Nil (4,295; 78.6%)	Dinoprostone Vaginal Suppository (930; 17%)	ARM (165; 3%)	Oxytocin (20; 0.4%)
	Balloon (53; 1%)			
Indication for induction	PRM (119; 22%)	SIUGR (58; 11%)	HTN (31; 6%)	Diabetes (147; 27%)
	Fetal distress (67; 13%)	PP (91; 17%)	Blood group isoimmunization/Chorioamnionitis/ Cholestasis (22; 4%)	
Re-induction	Yes (45; 3%)	No (1,741; 97%)		
Pain relief	Nil (2,395, 44%)	Non-pharma (602; 11%)	Pethidine (1,257; 23%)	Nitrous oxide (508; 9%)
	Non-pharma and Entonox (160; 3%)	Pethidine and Entonox (541; 10%)		
Ruptured membrane ^†^	Spontaneous (2,490; 58%)	Artificial (1,778; 42%)		
Amniotic fluid liquor	Nil (31; 1%)	Clear (3,528; 86%)	Scanty (4; 0.1%)	
	Meconium (465; 11%)	Blood stained (53; 1%)		
Type of labor	Spontaneous (2,221; 45%)	Augmented (788; 16%)	Induced (846; 17%)	No labor (1,067; 22%)
Estimated blood loss^†^	< 500 ml (3,537; 65%)	500–999 ml (1,586; 29%)	1,000–2,000 ml (284; 5.5%)	> 2,000 ml (24; 0.5%)
Complications†	Antepartum hemorrhage (25; 0.5%)	Postpartum hemorrhage (151; 3%)	Placental abruption (45; 1%)	Placenta previa (47; 1%)
	Shoulder dystocia (26; 0.5%)	Cord prolapse (8; 0.2%)	Hysterectomy (5; 0.1%)	Ruptured uterus (3; 0.1%)
Placenta complete^†^	Yes (5,008; 96%)	No (187; 4%)		
Strep B	Yes (782; 14%)			
Blood transfusion	Yes (84; 1.5%)			
Rh negative	Yes (194; 4%)			
Tubal Ligation	Yes (73; 1.3%)			
Stem cell collected	Yes (208; 4%)			

Abbreviations: ARM, artificial rupture of the membrane; BP, blood pressure; CS, cesarean section; HTN, hypertension; IDDM, insulin-dependent diabetes mellitus; NIDDM, noninsulin-dependent diabetes mellitus; NVD, normal vaginal delivery; PP, prolonged pregnancy; PRM, premature rupture of the membrane; SIUGR, suspected intrauterine growth restriction; UAE, The United Arab Emirates.

**Fig. 1:**
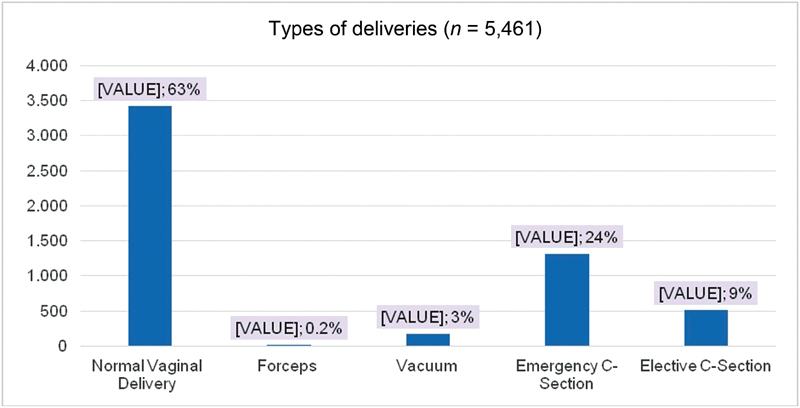
Types of deliveries are presented in number and percentage.

The birth indication rate was: 64% NVD, 24% emergency CS, 9% elective CS, 3% vacuum, and 0.3% forceps (Fig. 1). The rate of vaginal birth after previous CS was 261 (6%), of external cephalic version was 28 (0.7%), and of induction was 1,168 (21.4%). Anesthesia was used in 1,806 (33%) cases. Artificial rupture of the membrane was performed in 1,778 (42%) of the women, and 522 (12%) of the women had amniotic fluid liquor (Table 1).

**Fig. 2:**
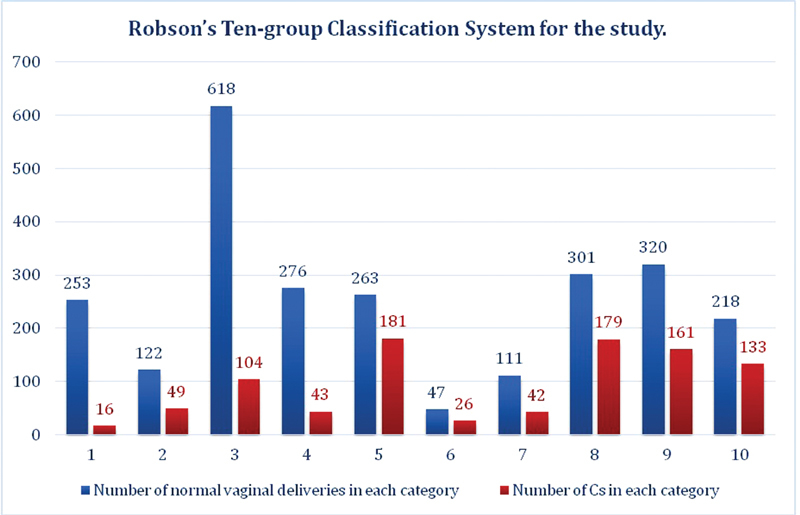
Robson 10-group classification system. The deliveries in Blue are normal vaginal deliveries, and those on orange are number of cesarean sections in each category.

Table 2 and Fig. 2 show number of CSs and its relative size in each group according to Robson classification. Multiparous women with previous CS (group 5), women with multiple pregnancies (group 9), and women with a single transverse or oblique lie (group 9), constituted the the largest groups in our study (*p* < 0.001).

**Table 2:** Robson 10-group classification system

No. of deliveries	*n = *5,461
**No. of CSs**	1,824; 33%
Robson categories*	
**Category 1**	253; 4.6%
**Category 1 with CS**	16; 1%
**Category 2**	122; 2%
**Category 2 with CS**	49; 3%
**Category 3**	618; 11%
**Category 3 with CS**	104; 6%
**Category 4**	276; 5%
**Category 4 with CS**	43; 2.4%
**Category 5**	263; 5%
**Category 5 with CS**	181; 10%
**Category 6**	47; 1%
**Category 6 with CS**	26; 1.4%
**Category 7**	111; 2%
**Category 7 with CS**	42; 2%
**Category 8**	301; 5.5%
**Category 8 with CS**	179; 9.8%
**Category 9**	320; 6%
**Category 9 with CS**	161; 9%
**Category 10**	218; 4%
**Category 10 with CS**	133; 7%

Abbreviation: CS, cesarean section.

* The ten categories are:

(1) Primiparous women with a single cephalic pregnancy ≥ 37 weeks of gestation, in spontaneous labor

(2) Primiparous women with a single cephalic pregnancy, ≥ 37 weeks of gestation, submitted to induction of labor or to CS prior to the onset of labor

(3) Multiparous women without a previous uterine scar, with a single cephalic pregnancy ≥ 37 weeks of gestation, in spontaneous labor

(4) Multiparous women without a previous uterine scar, with a single cephalic pregnancy ≥ 37 weeks of gestation, submitted to induction of labor or to CS prior to the onset of labor

(5) Multiparous women with 1 or more previous uterine scar(s) and a single cephalic pregnancy ≥ 37 weeks of gestation

(6) Primiparous women with a single breech pregnancy

(7) Multiparous women with a single breech pregnancy, with/without previous uterine scar(s)

(8) Women with multiple pregnancies with/without previous uterine scar(s)

(9) Women with a single pregnancy with a transverse or oblique lie, with/without previous uterine scar(s)

(10) Women with a single cephalic pregnancy at ≤ 36 weeks of gestation

The antenatal history showed that 993 (18%) of the women had gestational diabetes mellitus (GDM), while 80 (2%), and 43 (1%) had type 1 and type 2 diabetes, respectively. In addition, 265 (5%) of the women had high blood pressure, and 73 (1%) had preeclampsia. The percentage of multiple gestations was 3.5% (1,824) of all pregnancies. The prevalence of non-vertex presentation was 15%, and the overall CS rate was 33% (1,824 out of 5,428), with 46.5% (849 out of 1,824) primary CS, and 53.5% (975 out of 1,824) repeated CS. Neonatal data showed that the majority of babies were boys (2,811; 51.4%), and that the delivery was led mostly by a doctor (2,809; 51.5%). The births were multiple in 177 (3.2%) of the pregnancies, with 3% having a breech presentation, and other 3% in other positions. We fit several multiple regression models to better understand the predictability of obstetrical interventions and outcomes (Table 3). Several variables were found to be strongly correlated with statistical significance. Table 4 depicts the multiple regression models of parity, birth indication, gestational age, perineal injury, and birth weight. All of the variables that were statistically significant predictors remained statistically significant at the same *p* values when the semipartial R^2^ was calculated. There were several significant predictors of maternal diabetes, of hypertension, and of birth weight (Table 5).

**Table 3:** Multiple regression models present the predictability of obstetrical interventions and outcomes. Statistically significant *p* (*p*< 0.05) is presented in bold

	Maternal age	Nationality	Parity	Gestational age/weeks	Diabetes	Hypertension	Previous cesarean section (CS)
**Nationality**	**0.0001**						
**Parity**	**0.0000**	**0.0000**					
**Gestational age/weeks**	**0.0001**	1.0000	1.0000				
**Diabetes**	**0.0000**	1.0000	**0.0009**	**0.0075**			
**Hypertension**	**0.0000**	1.0000	1.0000	**0.0000**	**0.0000**		
**Previous CS**	**0.0000**	0.2011	**0.0000**	**0.0000**	**0.0000**	1.0000	
**Birth indication**	**0.0000**	**0.0289**	1.0000	**0.0000**	**0.0000**	**0.0000**	**0.0000**
**Anesthesia**	**0.0000**	1.0000	**0.0001**	**0.0000**	**0.0000**	**0.0000**	**0.0000**
**Perineal injury**	**0.0000**	**0.0000**	**0.0000**	**0.0000**	**0.0000**	**0.0065**	**0.0000**
**Birth Weight/g**	1.0000	0.7611	**0.0000**	**0.0000**	1.0000	**0.0000**	**0.0009**
**Vaginal Birth After Cesarean (VBAC)**	**0.005**	1.0000	**0.0000**	1.0000	1.0000	1.0000	**0.0000**

**Table 4:** Multiple regression and semipartial correlations of parity, birth indication, gestational age, perineal injury, and birth weight show variables that were statistically significant predictors. These predictors remained statistically significant when we calculated the semipartial R2. Statistically significant *p* (*p* < 0.05) presented in bold. Only significant results are displayed

	Coefficient	Standard error	t	*p*-value	95% CI	Semipartial R^2^ and its *p*-value
**Parity (*n* = 5016, F (14,5001) = 206.12, Eta Sq^†^ = 0.365 Omega Sq^‡^ = 0.364)**						
**Maternal age**	.2227481	.0083859	26.56	**0.000**	.2063079 0.2391882	0.0895 **0.0000**
**Nationality**	−.0819406	.0133661	−6.13	**0.000**	−.1081441 −0.0557372	0.0048 **0.0000**
**Previous CS**	.1933498	.0096646	20.01	**0.000**	.1744029 0.2122967	0.0507 **0.0000**
**Booked**	.1193474	.0256029	4.66	**0.000**	.0691545 0.1695404	0.0028 **0.0000**
**Birth indication (*n* = 5016, F (14,5001) = 314.98, Eta Sq^†^ = 0.4686 Omega Sq^‡^ = 0.4671)**						
**Maternal age**	.085875	.0219318	3.92	**0.000**	.0428791 0.128871	0.0016 **0.0001**
**Nationality**	.2063516	.0327676	6.30	**0.000**	.1421127 0.2705906	0.0042 **0.0000**
**Parity**	−8075477	.0327394	−24.67	**0.000**	−.8717313 −0.7433641	0.0647 **0.0000**
**Gestational age/weeks**	−1438536	.0421987	−3.41	**0.001**	−.2265816 −0.0611257	0.0012 **0.0007**
**Diabetes**	−.1065637	.0302752	−3.52	**0.000**	−.1659163 −0.0472111	0.0013 **0.0004**
**Hypertension**	−.1147106	.0367175	−3.12	**0.002**	−.186693 −0.0427283	0.0010 **0.0018**
**Previous CS**	.9132178	.0209711	43.55	**0.000**	.8721052 0.9543303	0.2015 **0.0000**
**Perineal Injury**	−.3564856	.0101073	−35.27	**0.000**	−.3763004 −0.3366708	0.1322 **0.0000**
**Gestational age/wks (*n* = 5016, F (14,5001) = 99.80, Eta Sq^†^ = 0.2184 Omega Sq^‡^ = 0.2162)**						
**Maternal age**	−.014594	.0073491	−1.99	**0.047**	−.0290019 −0.0001868	0.0006 **0.0471**
**Nationality**	.0309317	.0110024	2.81	**0.005**	.0093622 0.0525012	0.0012 **0.0050**
**Parity**	.0350506	.0115951	3.02	**0.003**	.0123192 0.057782	0.0014 **0.0025**
**Diabetes**	.0282956	.010138	2.79	**0.005**	.0084206 0.0481706	0.0012 **0.0053**
**Hypertension**	.0618182	.0122706	5.04	**0.000**	.0377624 0.0858739	0.0040 **0.0000**
**Previous CS**	−.054047	.0082078	−6.58	**0.000**	−.0701379 −0.0379562	0.0068 **0.0000**
**Booked**	−.215666	.0208366	−10.35	**0.000**	−.2565157 −0.1748181	0.0167 **0.0000**
**Birth indication**	−.016116	.0047276	−3.41	**0.001**	−.0253841 −0.006848	0.0018 **0.0007**
**Birth weight/g**	.3285245	.0116475	28.21	**0.000**	.3056903 0.3513587	0.1243 **0.0000**
**Birth weight/g (*n* = 5016, F (14,5001) = 73.63, Eta Sq^†^ = 0.1709 Omega Sq^‡^ = 0.1686)**						
**Nationality**	.0415472	.012402	3.35	**0.001**	.017232 0.0658624	0.0019 **0.0008**
**Parity**	.0716517	.013048	5.49	**0.000**	.0460718 0.0972317	0.0050 **0.0000**
**Gestational age/weeks**	.4177657	.014811	28.21	**0.000**	.3887287 0.4468026	0.1319 **0.0000**
**Diabetes**	−.033661	.011431	−2.94	**0.003**	−.0560723 −0.0112513	0.0014 **0.0032**
**Hypertension**	.0387665	.013861	2.80	**0.005**	.011592 0.065941	0.0013 **0.0052**
**Perineum Injury (*n* = 5016, F (14,5001) = 193.69, Eta Sq^†^ = 0.3516 Omega Sq^‡^ = 0.3498)**						
**Maternal age**	−.071298	.027481	−2.59	**0.010**	−.1251752 −0.0174224	0.0009 **0.0095**
**Nationality**	.1789769	.041109	4.35	**0.000**	.0983851 0.2595686	0.0025 **0.0000**
**Parity**	−1.17556	.040102	−29.31	**0.000**	−1.254185 −1.09695	0.1114 **0.0000**
**Gestational age/weeks**	.2726134	.052752	5.17	**0.000**	.169195 0.3760318	0.0035 **0.0000**
**Previous CS**	.2742744	.030589	8.97	**0.000**	.2143063 0.3342426	0.0104 **0.0000**
**Birth indication**	−.558777	.015842	−35.27	**0.000**	−.5898361 −0.5277182	0.1613 **0.0000**
**Birth weight/g**	.1236014	.046872	2.64	**0.008**	.031711 0.2154919	0.0009 **0.0084**

Abbreviation: CS, cesarean section *t* ^∗^, distribution; ^∗∗^semipartial regression; ^†^eta squared is an effect size measure for one-way or factorial ANOVA; ^‡^Omega squared (ω2) is a measure of effect size, or the degree of association for a population.

**Table 5:** Multiple regression and semipartial correlations of diabetes and hypertension show variables that were statistically significant predictors. Those predictors remained statistically significant when we calculated the semipartial R2. Statistically significant *p* (*p* < 0.05) presented in bold. Only significant results are displayed

	Coefficient	Standard error	t	*p*-value	95% CI	Semipartial R^2^ and its *p*-value
Diabetes						
**Hypertension**	.1074387	.017077	6.29	**0.000**	.0739587 0.1409187	0.0075 **0.0000**
**Maternal age**	−.088283	.010170	−8.68	**0.000**	−.1082219 −0.0683449	0.0143 **0.0000**
**Hypertension (** ***n*** ** = 5016, F (14,5001)** **= 13.13, Eta Sq** **= 0.0354 Omega Sq** **= 0.0327)**						
**Maternal age**	−.047316	.008424	−5.62	**0.000**	−.0638325 −0.0308007	0.0061 **0.0000**
**Gestational age/weeks**	.0816826	.016213	5.04	**0.000**	.0498969 0.1134684	0.0049 **0.0000**
**Diabetes**	.0730829	.011616	6.29	**0.000**	.0503089 0.095857	0.0076 **0.0000**
**Previous CS**	.0210824	.009470	2.23	**0.026**	.0025152 0.0396496	0.0010 **0.0261**

Abbreviation: CS, cesarean section. *t*-distribution; ^∗∗^semipartial regression; ^†^eta squared is an effect size measure for one-way or factorial ANOVA; ^‡^Omega squared (ω2) is a measure of effect size, or the degree of association for a population.

## Discussion

Health is one of the key indicators of socioeconomic development in society; and maternal health is one of the vital health indicators in any country.^13^ It is well known that the continuum of care has become a core strategy for reducing maternal, newborn, and child mortality by promoting integrated maternal and neonatal health services. Over the past few decades, the steady rise in the CS rates have led to an increased concern among healthcare professionals, governments, policymakers, and clinicians.^1^ ^3^ ^13^ Several factors, such as the awareness of pregnant women regarding NVD, the deficiency of knowledge about the complications of CSs, the fear of NVD, and the reduced role of midwives in maternity hospitals, have led to an increased inclination by part of the mothers to undergo a CS.^14^ Furthermore, other factors, such as maternal age, progress in surgical techniques, social and economic factors, health insurance coverage, and lack of proper training during the pregnancy, have led to a decreased willingness to undergo a NVD.^15^

The analysis of more than 5,400 deliveries (60% of all the deliveries in 2016) in 2 tertiary care maternity hospitals in Dubai showed that groups 5, 8 and 9 of Robson 10-group classification system were the largest contributors to the overall CS rate and accounted for 30% of the total CS rate (Table 2). The average global CS rate shows the lowest CS rates in Africa (7.3%), and the highest CS rates in Latin America and in the Caribbean (40.5%), while Asia is in the middle, with a CS rate of 19.2%.^15^ The results of the present study show that the CS rate in the UAE is higher than the global average CS rate, as well as than the average CS rate in Asia, which highlights the need for more education of pregnant women and of their physicians to promote NVD. Several resource-rich countries have responded to the public health concern posed by high CS rates by implementing policies designed to increase the NVD rates.^16^ ^17^ Reducing a relatively high CS rate is a long-term achievement, which requires a stringent plan over several years, involving several staff categories. However, the decrease in the CS rate might have some negative consequences, such as an increase in the number of forceps deliveries and of obstetric anal sphincter injuries, as well as in the number of newborn babies with low Apgar scores.^18^

The results of the present study show that we need to propose and evaluate interventions for the improvement of labor management in women with multiple pregnancies, as well as in those with a single pregnancy with a transverse or oblique lie, and to promote vaginal delivery after previous cesarean section to mitigate further increases in the future. Several studies showed that the main reason of CS is the previous cesarean section.^19^ The scientific, public health, and medical communities have raised concern about this global epidemic, while the search for ideas and interventions to reduce unnecessary CSs is ongoing.^10^ ^20^ Hence, a proper planning is needed to reduce the number of CSs in nulliparous women in order to prevent repeated CSs in the future. Fear of NVD, the age of the mother and the recommendation of the physician are the most influential factors that encourage mothers to undergo a CS. Therefore, holding consultation sessions before and during the pregnancy could help mothers to choose the best method of delivery.^21^ Furthermore, familiarity with the delivery room, staff, equipment, analgesia, presence of visitors, and making the delivery room pleasant^22^ are factors that reduce maternal anxiety and aid the mother in choosing the best method of delivery.

However, the reasonable and responsible reduction of unnecessary CSs is not a trivial task, and it will take considerable time and effort. Monitoring both the CS rates and outcomes is essential to ensure that policies, practices, and actions for the optimization of the utilization of CS lead to improved maternal and infant outcomes. Nonetheless, the present article is an observational retrospective study, based on routinely collected data with an explorative character, and it does not allow for causal explanations. The strength of the present study, given the fact that data were abstracted for > 5,000 women who presented to tertiary care hospitals in Dubai, far outweighs its limitation. It is anticipated that the results of our study will encourage healthcare providers, policy makers, and decision makers to establish a more decisive policy to encourage NVD in comparison to CS.
